# Growth Hormone Overexpression Induces Hyperphagia and Intestinal Morphophysiological Adaptations to Improve Nutrient Uptake in Zebrafish

**DOI:** 10.3389/fphys.2021.723853

**Published:** 2021-09-01

**Authors:** Marcela G. Meirelles, Bruna F. Nornberg, Tony L. R. da Silveira, Mateus T. Kütter, Caroline G. Castro, Juan Rafael B. Ramirez, Virgínia Pedrosa, Luis Alberto Romano, Luis Fernando Marins

**Affiliations:** ^1^Laboratório de Biologia Molecular, Instituto de Ciências Biológicas, Departamento de Ciências Fisiológicas, Universidade Federal do Rio Grande – FURG, Rio Grande, Brazil; ^2^Laboratório de Bioquímica Funcional de Organismos Aquáticos, Instituto de Oceanografia, Estação Marinha de Aquicultura, Universidade Federal do Rio Grande – FURG, Rio Grande, Brazil; ^3^Laboratório de Imunologia e Patologia de Organismos Aquáticos, Instituto de Oceanografia, Estação Marinha de Aquicultura, Universidade Federal do Rio Grande – FURG, Rio Grande, Brazil

**Keywords:** *gh*-transgenic fish, *Danio rerio*, peptide transporter, feed intake, intestinal morphology

## Abstract

The excess of circulating growth hormone (GH) in most transgenic animals implies mandatory growth resulting in higher metabolic demand. Considering that the intestine is the main organ responsible for the digestion, absorption, and direction of dietary nutrients to other tissues, this study aimed to investigate the mechanisms by which *gh* overexpression modulates the intestine to support higher growth. For this purpose, we designed an 8-weeks feeding trial to evaluate growth parameters, feed intake, and intestinal morphometric indices in the adult *gh*-transgenic zebrafish (*Danio rerio*) model. To access the sensitivity of the intestine to the excess of circulating GH, the messenger RNA (mRNA) expression of intestine GH receptors (GHRs) (*ghra* and *ghrb*) was analyzed. In addition, the expression of insulin-like growth factor 1a (*igf1a*) and genes encoding for di and tripeptide transporters (*pept1a* and *pept1b*) were assessed. Gh-transgenic zebrafish had better growth performance and higher feed intake compared to non-transgenic sibling controls. Chronic excess of GH upregulates the expression of its cognate receptor (*ghrb*) and the main growth factor related to trophic effects in the intestine (*igf1a*). Moreover, transgenic zebrafish showed an increased intestinal absorptive area and higher expression of crucial genes related to the absorption of products from meal protein degradation. These results reinforce the ability of GH to modulate intestinal morphology and the mechanisms of assimilation of nutrients to sustain the energy demand for the continuous growth induced by the excess of circulating GH.

## Graphical Abstract

GH overexpression increases the intestinal mass and absorptive surface area both through the direct effects of GH transgenics (up-regulating the expression of its cognate receptor and insulin-like growth factor, *igf1a*) and through indirect effects through an increase in the amount of food consumed. In addition, hyperphagia provides a greater amount of nutrients in the intestinal lumen and stimulates the synthesis of di- and tri-peptide transporters, which are the main route of absorption of dietary products from protein degradation.

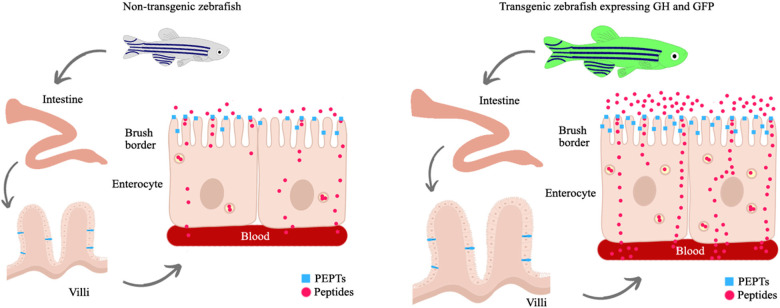

## Introduction

Growth in fish is regulated through complex interactions among multiple organs and hormones that form the somatotrophic axis (White et al., 2016). This axis consists of growth hormone (GH), insulin-like growth factors (IGFs), and their corresponding membrane receptors and binding proteins (Fuentes et al., 2013; Ranke and Wit, 2018). However, the hormones that make up the somatotrophic axis have other functions that are not directly related to growth. GH in humans, for example, can act directly on sexual maturation, immune and cardiovascular systems, appetite control, neurogenesis, lipid, carbohydrate and mineral metabolism, amino acid absorption, and nitrogen retention, in addition to affecting aging (for review refer (Lu et al., 2019)). To study the pleiotropic effects of GH, in 2007, the research group produced a transgenic zebrafish line (*Danio rerio*) that ubiquitously overexpresses GH (Figueiredo et al., 2007). Since then, we have been studying the effects of this overexpression on different biological systems of this model. It was shown that *gh*-transgenic zebrafish grew faster in a shorter period than their non-transgenic full sibling controls (Figueiredo et al., 2007; Rosa et al., 2010; Silva et al., 2015) and presented enhanced muscle hypertrophy when compared to non-transgenic fish (Kuradomi et al., 2011). GH overexpression also impacts oxidative metabolism (Rosa et al., 2008, 2011), aging (Rosa et al., 2010), reproduction (Figueiredo et al., 2013), ionic balance (Almeida et al., 2013), immune system (Batista et al., 2014), and appetite behavior (Dalmolin et al., 2015) in this *gh*-transgenic zebrafish line. Collectively, these findings provide evidence that the excess of circulating GH implies mandatory growth even in unfavorable conditions, resulting in higher metabolic demand.

Metabolic reorganization occurs in GH transgenic fish to meet their increased energy demands to improve growth and other physiological processes (Leggatt et al., 2009; White et al., 2016). It has long been known that the size, structure, and functional properties of the gastrointestinal (GI) tract of vertebrates may change to match the current functional demands (Starck, 2003). As the organ with one of the largest surface areas facing the environment (Spanier and Rohm, 2018), the intestine houses over 22 digestive enzymes and expresses over 53 channels, and transports proteins in its brush-border membrane (McConnell et al., 2011) for nutrient uptake. The dietary nutrients are essential for the construction of living tissues and they also are a source to supply energy for growth, reproduction, and health (Jia et al., 2017). In this regard, previous studies indicated that the growth and feed utilization of *gh*-transgenic salmon (Raven et al., 2006; Leggatt et al., 2009) and mice (Ulshen et al., 1993) were related to intestinal function. GH transgenesis influences differentially the utilization of dietary macronutrients (carbohydrates, protein, and lipids) for energy production (Leggatt et al., 2009). In the same way, it has been proposed that GH treatment enhanced transport/absorption of amino acids by the intestine of teleosts (Collie and Stevens, 1985; Sun and Farmanfarmaian, 1992; Farmanfarmaian and Sun, 1999; Walker et al., 2004), being these molecules the major energy substrates for the tissue of zebrafish (Jia et al., 2017). For teleosts, it has been proposed that 14–85% of the energy requirements is provided by amino acids depending on the stage of development (Van Waarde, 1983; Jia et al., 2017).

Although the transport of amino acids *via* the enterocyte plasma membrane is mediated by several classes of amino acid transporters, the transport of di and tripeptides is mediated by a single carrier system, called PEPT1 (Peptide Transporter 1) or SLC15A1 (solute carrier 15 family member A1) (for a review refer 27). In the 1990s, the intestinal oligopeptide transporter PEPT1 was cloned and characterized by various eukaryotes [reviewed by Spanier and Rohm (2018)]. Verri et al. (2003) described the molecular and functional characterization of the zebrafish PEPT1 transporter (Verri et al., 2003). As in mammals, PEPT1 is abundantly expressed particularly in the proximal intestine and provides highly effective transport of small peptides across the brush-border membrane of enterocytes (Verri et al., 2003, 2017; Vacca et al., 2019). The expression levels and functional activities of peptide transporters are affected by various factors, such as dietary composition, nutrient supply, developmental stage, physiological status, and hormones (Rubio-Aliaga and Daniel, 2008; Spanier, 2014; Spanier and Rohm, 2018), especially regarding the intestine. Significant effects of selected hormones on PEPT1 have been shown in the last decade. In mice, intestine-specific depletion of leptin signaling substantially reduced di and tripeptides uptake in the intestine (Tavernier et al., 2014). Insulin is also associated with the regulation of PEPT1 expression and function in rats and humans (Bikhazi et al., 2004). In fishes, there is evidence that leptin, cholecystokinin, and gastrin may have regulatory effects in intestinal peptide transport (Ostaszewska et al., 2010a, b; Koven and Schulte, 2012), but there is little information on the hormonal control of peptide absorption caused by supraphysiological levels of GH.

Since PEPT1 is the major route for the absorption of products from protein degradation after a meal (Spanier, 2014), many studies have investigated the functional plasticity of this class of proteins and how they functionally (co)operate to support growth in different animal models [for a review, refer Verri et al. (2017)]. Zebrafish is a model organism with extensive literature regarding their stomachless gut morphology and digestive tract development [reviewed by Wang et al. (2010) and Brugman (2016)]. While the human GI tract is divided into stomach, duodenum, jejunum, ileum, and colon, the zebrafish GI tract is generally subdivided into three distinct regions: intestinal bulb, midgut, and hindgut (Wang et al., 2010). However, how hormones such as GH interact with the GI tract still need to be studied. For this purpose, *gh*-transgenic fishes provide a powerful model to understand how chronic GH excess could regulate morphophysiological mechanisms in the intestine to maintain a higher growth rate and energy requirement. Thus, this study aimed to investigate the effects of GH overexpression on intestinal morphology, ultrastructure, and peptide transport in a transgenic zebrafish line.

## Materials and Methods

### Animals and Conditions

**FIGURE 1:**
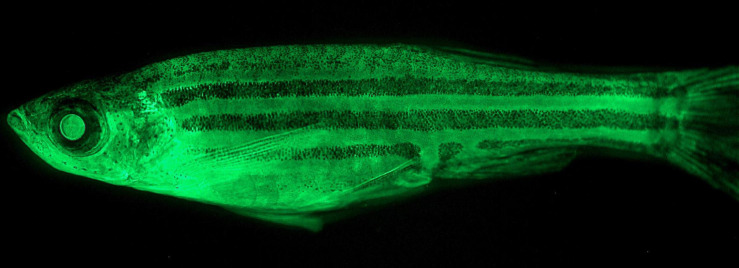
*Gh*-transgenic zebrafish (*Danio rerio*). The F0104 line was developed from the co-injection of two separate transgenes, both containing the carp β-actin promoter (*Cyprinus carpio*), which controls the expression of the GFP gene from the jellyfish *Aequorea victoria* or the GH gene from the marine silverside fish (*Odonthestes argentinensis*). Linearized transgenes were co-injected in an equimolar ratio into zebrafish zygotes to provide the same genome integration and expression (Figueiredo et al., 2007). In the F0104 line, GFP expression works not only as a marker of transgenesis but also allows mapping the tissues that are expressing GH since both GFP and GH are under the control of the same gene promoter.

The *gh*-transgenic zebrafish line used in this study was generated by Figueiredo et al. (2007). The transgenic and non-transgenic zebrafish were obtained by crossbreeding between the F0104 line and wild zebrafish. In addition to *gh* transgene, the *gh*-transgenic zebrafish expresses the green fluorescent protein (GFP) as a transgenesis tag (Figure 1). The non-transgenic zebrafish were siblings of the transgenic zebrafish which did not incorporate the genetic construct into their genome. All the procedures involving animals were conducted in accordance with the Brazilian Guidelines for the Care and Use of Animals for Scientific and Educational Purposes and were approved by the Ethics Committee of the Federal University of Rio Grande (FURG) – Brazil (Protocol: 23116.008403/2018-32).

*Gh*-transgenic and non-transgenic zebrafish groups were kept in a recirculating aquaculture system. A total of 80 zebrafish 7-month-old, of both sexes (40 transgenic and 40 non-transgenic full siblings) were raised in eight tanks containing 15 L of water (four tanks/group and 10 animals/tank). *Gh*-transgenic zebrafish co-expressing GFP were identified under an epifluorescence microscope and placed in a separate tank. The tanks were connected to a filter tank filled with zeolite, bioceramic, and ultraviolet (UV) lamp, and a heater thermostat. The photoperiod was adjusted to 14:10 h, light/dark period. Management and maintenance of zebrafish complied with the Zebrafish Book^1^. During the breeding period, the water temperature was maintained at 27.2 ± 0.3°C. The water pH (7.5 ± 0.1) and dissolved oxygen (>6 mg L^–1^) were measured daily. Water quality parameters were measured once a week using commercial kits (Labcon Ammonia Alcon Fresh Water; Labcon Test Nitrite Alcon, Brazil). Total ammonia and nitrite levels were maintained lower than 0.5 and 0.25 mg L^–1^, respectively. The tanks were scrubbed, debris and feces siphoned out, and 25% of the water changed every 3 days. Until the start of the experiment, fish were fed two times a day (5 days a week) *ad libitum* with commercial feed (Tetra ColorBits, Germany, 47.5% crude protein). For the analysis of feed intake, a predetermined amount of feed was weighed and offered in small portions to the fish until they stopped feeding for at least 2 min. The remaining feed was weighed again and the difference was considered as feed consumed. This approach allowed us to estimate the amount of feed that a group of fish was able to ingest at each feeding event during the experimental period.

### Growth Parameters and Tissue Collection

From day 0 to day 60, every 15 days, fish were anesthetized (buffered tricaine methanesulfonate MS-222, pH 7.2, Sigma-Aldrich, United States, 100 mg L^–1^), individually weighed (mg) and measured in lateral decubitus for standard length (cm). Throughout the 60-day feeding trial, animals were hand-fed to apparent satiety three times a day. At the end of the experiment, fish were euthanized using an overdose of buffered MS-222 at 400 mg L^–1^. Fish were then dissected and samples of the whole intestine were individually removed, weighed (mg), and measured in length (cm). After this, samples were immediately stored for histological and gene expression analysis. For tissue fixation, the fish were fasted for 24 h to clean the intestinal contents. It should be mentioned in this study that the sampling was performed in a single day. The whole process took <5 h.

Growth performance indicators were measured, such as weight gain (mg), standard body length (cm), feed intake (mg), specific growth rate (SGR), enterosomatic index (ESI), and intestinal quotient (IQ), using the following formulas:

Weight gain = Final weight − Initial weight;
SGR = ([Ln Final weight − Ln Initial weight/*t*] × 100);
ESI = ([intestinal weight/body mass] × 100);
IQ = ([intestinal length/standard length] × 100).

### Histological Analysis

**FIGURE 2:**
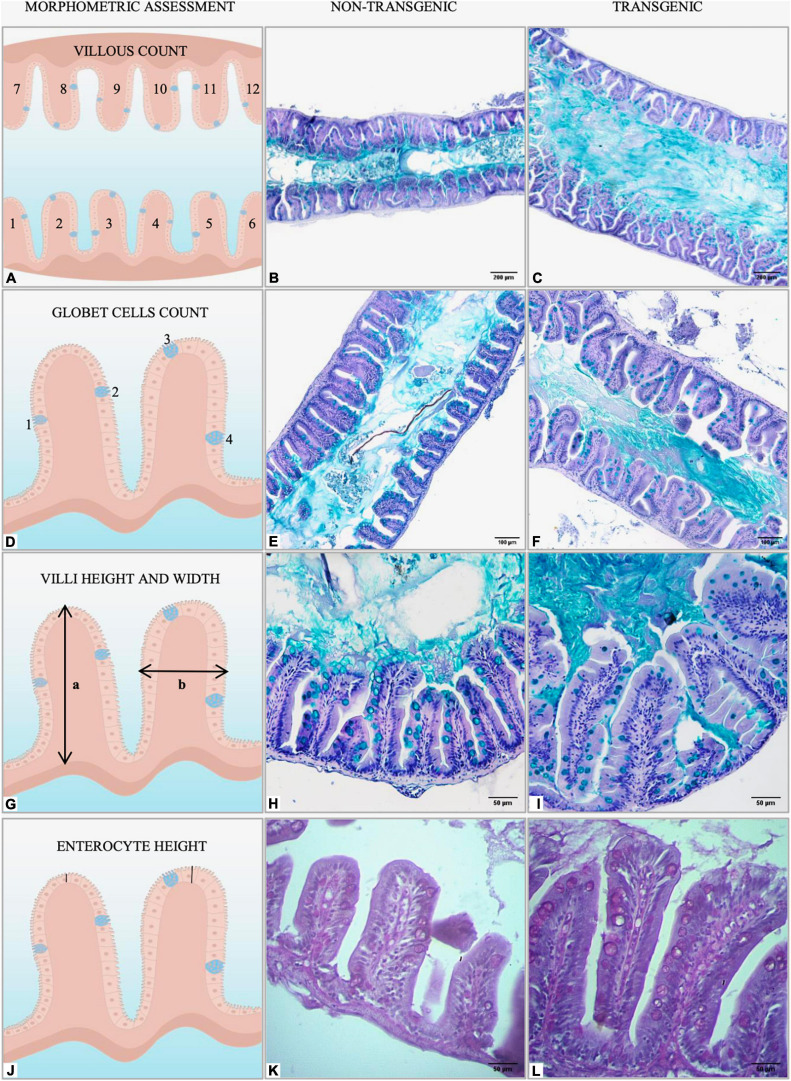
Graphical representation of morphometric analysis performed in the intestine of non-transgenic and *gh*-transgenic zebrafish (*D. rerio*) siblings. **(A)** Intestinal folds were manually counted in six fields randomly sampled for each animal using the multipoint tool from ImageJ. Representative Alcian blue-stained sections of midgut cross-sections from **(B)** non-transgenic and **(C)** *gh*-transgenic zebrafish. Magnification: ×10, scale bar: 200 μm. **(D)** The number of intestinal goblet cells was determined by manually counting all cells in six randomly sampled fields for each animal using the multipoint tool from ImageJ. Representative images of midgut sections stained for the goblet cell marker Alcian blue from **(E)** non-transgenic and **(F)** *gh*-transgenic zebrafish. Magnification: ×20, scale bar: 100 μm. **(G)** Intestinal villus height (a) and width (b) were manually measured in the anterior and middle intestine from 24 individual villi randomly sampled for each animal using the segmented line tool from ImageJ. Representative Alcian blue-stained sections of anterior intestine from **(H)** non-transgenic and **(I)** *gh*-transgenic zebrafish. Magnification: ×40, scale bar: 50 μm. **(J)** Enterocyte height was manually measured in the anterior intestine from 24 individual villi randomly sampled for each animal using the segmented line tool from ImageJ. Representative Periodic acid-Schiff (PAS) stained sections of anterior intestine from **(K)** non-transgenic and **(L)** *gh*-transgenic zebrafish. Magnification: ×40, scale bar: 50 μm, *n* = 18 non-transgenic and 18 *gh*-transgenic zebrafish.

Intestines (*n* = 18 samples per group) were histologically evaluated in paraffin-embedded tissue sections stained with Periodic acid-Schiff (PAS), Alcian blue, and hematoxylin and eosin (H&E). For morphometry, longitudinal sections (about 5 μm) were evaluated using brightfield microscopy (Primo Star, Zeiss, Germany) and the micrographs obtained with the Zen software (Axion Vision 4.8.2.0, Carl Zeiss, Germany). Intestinal folds were counted in the entire intestine in six fields randomly sampled on PAS-stained slides (×10 magnification, Figure 2A). Representative intestinal goblet cell number was determined by counting cells in the anterior and middle intestine in six fields randomly sampled on Alcian blue-stained slides (×20 magnification, Figure 2B). Mean villus height and width (×40 magnification, Figure 2C) and mean enterocyte length (×40 magnification, Figures 2D,D), were measured in the anterior and middle intestine on PAS-stained slides with a minimum of 24 villi averaged per fish to make *n* = 1. All the measurements were taken from micrographs using Software ImageJ (Abràmo et al., 2004) and performed by calibrated and blinded analyzers.

### Morphometric Analysis by Transmission Electron Microscopy

For morphometric transmission electron microscopy (TEM) analysis, the anterior segment of each intestine was used since this region is considered to be the main site of nutrient absorption in teleost fishes (Løkka et al., 2013; Faccioli et al., 2016). Fragments of the anterior intestine (*n* = 6 samples per group) were fixed for 48 h at 4°C in a solution of 6% paraformaldehyde, 3% glutaraldehyde in phosphate buffer (pH 7.4) to analyze the morphometry of intestinal microvilli. The samples were then post-fixed for 2 h in 2% osmium tetroxide + 0.4 M sodium cacodylate (pH 7.4, in the proportion of 1:1), dehydrated in a graded ethanol and acetone series, and later embedded in Araldite resin. Ultra-thin sections (60–80 nm) were mounted on copper grids and contrasted with uranyl acetate and lead citrate (Reynold’s solution). Analysis and photographic documentation were performed with an EM 900 ZEISS transmission electron microscope (Zeiss, Germany).

Images of intestinal epithelium (*n* = 15 micrographs of each sample) were used for measurements of height (*H*) and diameter (*D*) of the microvilli (for a total of 225 microvilli averaged per fish to make *n* = 1), using the Software ImageJ (Abràmo et al., 2004). With the above data, the microvilli surface area was calculated in the following formula: μm^2^ = ([*H*π*D*] + [π*R*^2^]), where *R* = 0.5D (Faccioli et al., 2016). Calibrated and blinded analyzers performed all measurements.

### RNA Extraction and Complementary DNA Synthesis

Nine individuals of each group were randomly sampled for gene expression analysis. Total RNA was extracted from the intestine using TRIzol Reagent (Invitrogen, Brazil), following the protocols from the manufacturer. RNA was treated with DNAse I Amplification Grade (Invitrogen, Brazil) to remove genomic DNA contamination. RNA quality and quantity were determined by spectrophotometry using a BioDrop (Isogen Life Science, B⋅V, Veldzigt, Netherlands). RNA integrity was assessed through electrophoresis on 1% agarose gel. Complementary DNA (cDNA) was synthesized from 1 μg of total RNA using a High Capacity cDNA Reverse Transcription (Applied Biosystems, São Paulo, Brazil) according to the recommendation from the manufacturer.

### Gene Expression Analyses by Quantitative Reverse Transcription Polymerase Chain Reaction

Quantitative reverse transcription polymerase chain reactions (qRT-PCRs) were performed with the SYBR green method in a 7500 Real-Time System (Applied Biosystems, MA, United States). The preliminary tests with standard dilution curves of cDNA showed that all primers used had efficiencies close to 98%. Each qRT-PCR reaction was performed in a 15 μl mixture contained 1.2 μl diluted cDNA, 7.5 μl Power-Up^TM^ SYBR Green Master Mix^TM^ (Thermo Fisher Scientific, São Paulo, Brazil), 0.375 μl forward and reverse each gene-specific primer, and 5.55 μl nuclease-free water. Duplicate PCRs were carried out for each sample. The amplification conditions were 95°C for 10 min, 40 cycles at 95°C for 15 s, and 60°C for 1 min. The melting curves of amplified products were generated to ensure the specificity of assays at the end of each qRT-PCR. In this study, three candidate reference genes (*elongation factor 1 alpha, eef1α*; β-*actin*, *actb1*; and *acidic ribosomal protein*, *rpl13a*) were tested using geNorm VBA applet for Microsoft Excel (Vandesompele et al., 2002). The most stable genes were *eef1α* and *rp113a*, which were selected as reference genes, in accordance with Rassier et al. (2020). The details on primer sequences and efficiency used in the present study are provided in Table 1. Reference and target genes expression was evaluated by the relative quantification method (Pfaffl, 2001).

**TABLE 1:** Gene-specific primers were used for quantitative reverse transcription polymerase chain reactions (qRT-PCR).

Gene	Primer sequence (5′-3′)	R^2^	Efficiency (%)	Amplicon Size (bp)	Accession no.
*igf1a*	F: CAGGCAAATCTCCACGATCTC	0.99	100	61	NM_131825.2
	R: TTTGGTGTCCTGGGAATATCTGT				
*Ghra*	F: TGCTGTGCGCTACAAAATGG	0.97	93	62	NM_001083578.1
	R: GCTTCTGCAAAGGCTGATAGAAA				
*Ghrb*	F: GAACTCAGAGTCCGGGCAAA	0,98	97	117	NM_001111081.2
	R: AAAGACCAGCACAGCCGTAA				
*slc15a1a*	F: GGC TTC GGT TCC TCC TAC AC	0.98	97	96	XM_021478814.1
	R: CGA GTT GGG CTG CAT GTC TT				
*slc15a1b*	F: GCA TCT ACG CAA AGC AGA GC	0.99	98	73	NM_198064.1
	R: ATG AGG GCA ACC ACC ATG AG				
*slc15a2*	F: CAC AGC CGG AGA AGT CAT GT	0.99	98	80	NM_001039828.1
	R: GAA CGG ATT TCA TGC TCG CC				
*actb1*	F: GCTGTTTTCCCCTCCATTGTT	0.99	97	60	NM_131031.2
	R: TCCCATGCCAACCATCACT				
*ef1α*	F: GGGCAAGGGCTCCTTCAA	0.99	99	54	NM_131263.1
	R: CGCTCGGCCTTCAGTTTG				
*rpl13a*	F: TCTGGAGGACTGTAAGAGGTATGC	0.99	99	148	NM_212784.1
	R: AGACGCACAATCTTGAGAGCAG				

### Data Analysis

All data are presented as mean ± SD. Data were tested for normality using Shapiro-Wilk’s test and homogeneity of variance using Levene’s and Bartlett’s tests. Data that did not meet the assumptions of variance and normality were log-transformed prior to further analysis. Body weight against time was linearly modeled, and the slopes of the two groups were statistically compared to determine growth differences. ANOVA with multiple comparisons was performed between genotypes at each time for weight gain and body length. A two-tailed independent *t*-test was performed in the other measured parameters between the two groups. Significant differences were determined with a *p* < 0.05. All statistical tests were performed using GraphPad Prism 7.0 (San Diego, CA, United States).

## Results

### Growth Performance

**FIGURE 3:**
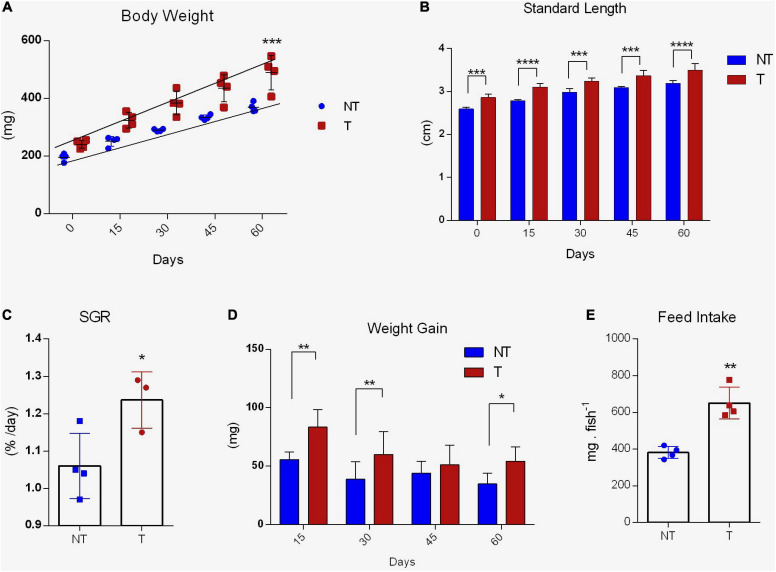
Growth performance and feed intake of *gh*-transgenic zebrafish (*D. rerio*). Body weight **(A)**, length **(B)**, SGR **(C)**, weight gain **(D)**, and feed intake **(E)** were significantly increased in response to GH excess in transgenic zebrafish compared to non-transgenic full siblings. Weight (mg) and length (mm) were measured every 15 days during the 60-day growth experiment (non-transgenic, *n* = 38, and *gh*-transgenic fish, *n* = 37). Differences in body weight between the two groups were determined by linear regression analysis. ANOVA with multiple comparisons was performed between the two genotypes at each time point for weight gain and body length. Comparisons of SGR and feed intake between two groups were taken by a two-tailed *t*-test. Data are shown as means ± SE of 37 fish per group. Asterisks indicate significant differences (*p < 0.05; **p < 0.01; ***p < 0.001; and ****p < 0.0001). Zebrafish that overexpress GH exhibited a linear increase in growth (*p* < 0.05). *gh*, growth hormone gene; NT, Non-Transgenic; T, Transgenic.

Over the 8-week growth analysis, significant growth response was detected in adult *gh*-transgenic zebrafish compared to non-transgenic siblings (Figure 3). The transgenic group expressed the phenotype attributed by the transgenesis, being statistically heavier (slopes 4.056 ± 0.40 and 2.862 ± 0.13; *R*^2^ 0.8472 and 0.9620, T and NT, respectively; *p* < 0.001) and longer (*p* < 0.0001) (Figures 3A,B, respectively) in comparison to the body mass and length of the non-transgenic group. At the start of the trial, non-transgenic and *gh*-transgenic adult zebrafish weighed 195.82 ± 6.31 mg and 240.95 ± 17.12 mg, while at the end of the experiment they weighted 369.07 ± 18.33 mg and 489.65 ± 24.55 mg, respectively (Figure 3A). The non-transgenic group presented 2.79 ± 0.03 cm and the transgenic group presented 3.15 ± 0.04 cm of length at the end of the experiment (Figure 3B). SGR showed a better performance in *gh* genotype compared to controls (1.237 ± 0.04 and 1.060 ± 0.04, respectively, *p* < 0.05) (Figure 3C). Additionally, repeated ANOVA measures detected differences in weight gain in favor of the transgenic group (*p* < 0.01). However, multiple comparisons did not detect differences in 45 days (Figure 3D). Feed intake was increased by 1.8-fold in transgenic group (*p* < 0.01) (Figure 3E). No significant mortalities were observed during the whole experiment (2 NT and 3 T fish).

### Intestinal Morphometrical Assessment

**FIGURE 4:**
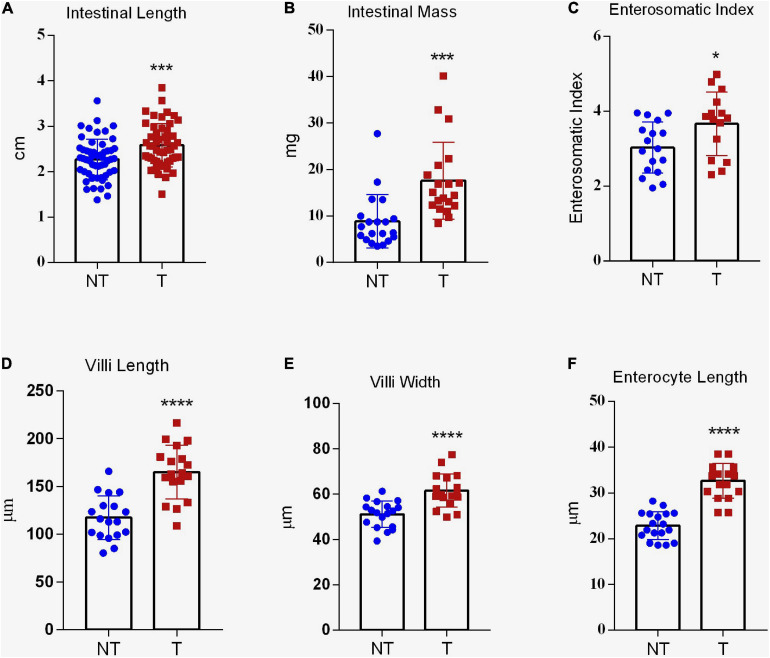
Intestinal absorptive area of *gh*-transgenic zebrafish (*D. rerio*). Intestinal length **(A)**, intestinal mass **(B)**, and enterosomatic index (ESI) **(C)** were significantly increased in response to GH excess in transgenic zebrafish compared to non-transgenic full siblings. Data are shown as means ± SE of 37 fish per group. *Gh*-transgenic zebrafish show a significantly enhancement of villus height **(D)**, villus width **(E)**, and length of enterocyte **(F)** with respect to the control. Differences in all intestinal parameters between the two groups were determined using a two-tailed *t*-test. Data are shown as mean ± SE of 18 fish per group. Asterisks indicate significant differences (*p < 0.05; ***p < 0.001; and ****p < 0.0001). NT, non-transgenic; T, transgenic.

*Gh*-transgenic zebrafish had longer intestine (*p* < 0.001) (Figure 4A). The non-transgenic group presented 2.36 ± 0.06 cm and the transgenic group presented 2.58 ± 0.06 cm of intestinal length. However, no significant difference was observed for IQ between the groups (data not shown), demonstrating that the increased intestine length in *gh* group was proportional to the increased in standard body length. Also, *gh*-transgenic fish had significantly heavier intestinal mass (NT = 9.35 ± 5.70 mg and *T* = 17.61 ± 8.28 mg, *p* < 0.001) and ESI (NT = 2.87 ± 0.66 mg and *T* = 3.61 ± 0.77 mg, *p* < 0.05) (Figures 4B,C, respectively).

As in mammals, the ultrastructure of the digestive tract has allowed us to infer about absorption of nutrients in fish (Zeng et al., 2014; Faccioli et al., 2016). In this sense, we investigate phenotypic modifications related to micro and ultrastructure in the intestine of the *gh*-transgenic zebrafish. In the histological assessment, villus length (*T* = 179.06 ± 27.42 μm and NT = 138.68 ± 22.43 μm; *p* < 0.0001), villus width (*T* = 62.93 ± 7.15 μm and NT = 54.44 ± 5.71 μm; *p* < 0.0001), and length of enterocyte (*T* = 36.63 ± 3.67 μm and NT = 22.12 ± 2.95 μm; *p* < 0.0001) were significantly higher in *gh*-transgenic zebrafish compared to those present in the intestine epithelium of control group (Figures 2H,I,K,L, 4D–F, respectively). However, the number of intestinal folds and goblet cells was not significantly affected (*p* > 0.05) (data not shown).

**FIGURE 5:**
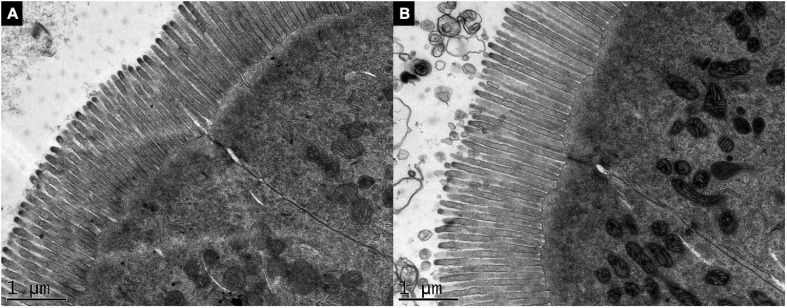
Ultrastructure of the intestinal microvilli of *gh*-transgenic zebrafish (*D. rerio*). Electron micrographs show organized microvilli on the apical surface in the enterocyte of non-transgenic **(A)** and *gh*-transgenic zebrafish **(B)** siblings. No difference in surface area of microvillus from the anterior intestine was detected between the groups (*p* > 0.05). Magnification: ×25.000, scale bar: 1 μm.

For the TEM analysis, the height and diameter of microvilli and the microvillus surface area did not vary between the groups (*p* > 0.05) (data not shown). Images showed no difference in the microvillus morphology in both intestine of control (Figure 5A) and transgenic fish (Figure 5B).

### Differential Gene Expression

**FIGURE 6:**
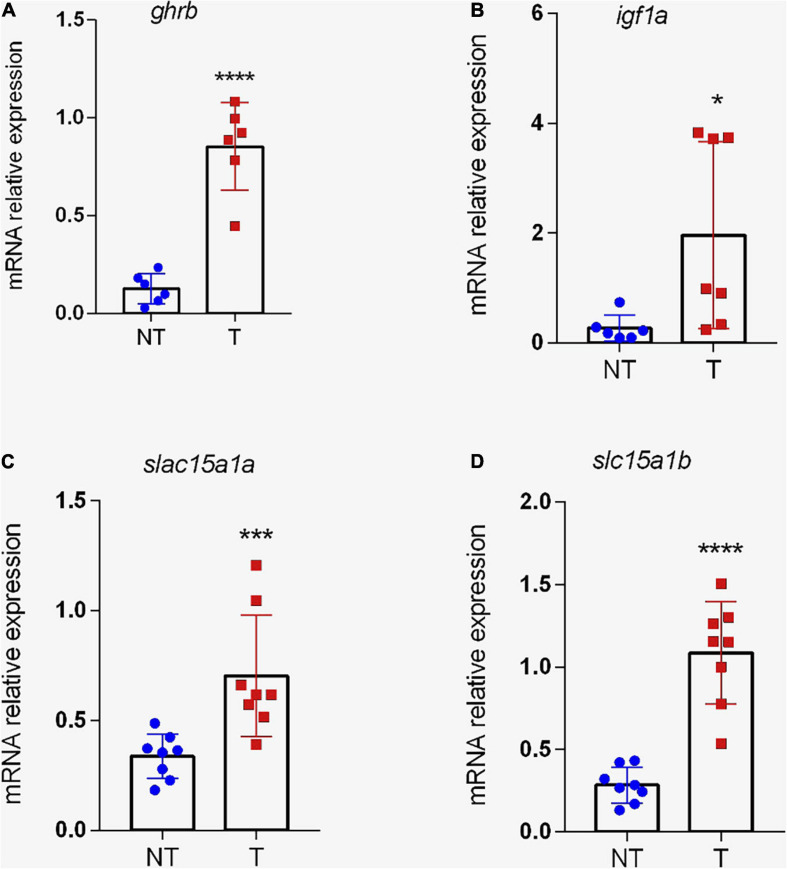
Expression of genes related to growth and peptide transport in the intestine of transgenic zebrafish (*D. rerio*). Relative *ghrb* **(A)**, *igf1a* **(B)**, *slc15a1a* **(C)**, and *slc15a1b* **(D)** gene expression normalized against *eef1α* and *rpl13a* in intestine from non-transgenic and *gh*-transgenic zebrafish. The transgenic group shows a significant increase in mRNA expression of *ghrb*, *igf1a*, and for the two genes that encode to PEPT1 transporter (*slc15a1a* and *slc15a1b)*. The differences in relative mRNA expression between the two groups were determined using a two-tailed *t*-test. Data are shown as mean ± SD. Asterisks indicate significant differences (*p < 0.05, ***p < 0.001; and ****p < 0.0001). NT, non-transgenic; T, transgenic.

To evaluate the intestine GH sensitivity of zebrafish, the expression of GHR was analyzed. Two paralogs of GHR have been defined in zebrafish based on their similarity: *ghra* and *ghrb* (Di Prinzio et al., 2010). Our data show that the transcriptional level of *ghrb* was significantly increased (*p* < 0.01) in the intestine of transgenic fish (Figure 6A), but the isoform *ghra* did not differ significantly (*p* > 0.05) between the groups (data not shown). Resembling *ghrb, igf1a* levels were higher (*p* < 0.05) in *gh*-transgenic intestine (Figure 6B).

Further, it was analyzed whether *gh* transgenesis could change the mRNA expression of genes related to transcellular transport of di and tripeptides in the intestine. According to Vacca et al. (2019), zebrafish PepT1a (*slc15a1a*) and PepT1b (*slc15a1b*) amino acid sequences shared 78% similarity and 62% identity. In this study, the levels of *slc15a1a* and *slc15a1b* transcripts were significantly increased (*p* < 0.01 and 0.0001, respectively) in *gh*-transgenic fish compared to controls (Figures 6C,D, respectively). No significant differences (*p* > 0.05) were noted in the transcriptional level of *slc15a2*.

## Discussion

In this study, we used a transgenic zebrafish model that overexpresses *gh* to investigate whether the excess of circulating GH could be related to changes in the morphology/ultrastructure of the intestine and molecular targets associated with absorption of dietary nutrients. Taking into account, it is already known that excess GH leads to an increase in food intake, we hypothesize that the intestine may adapt to this situation not only by changing its size but also by increasing the number of small peptide transporters, such as a strategy to increase energy uptake imposed by excess circulating GH.

Even though zebrafish has limited growth (Mommsen, 2001; Biga and Goetz, 2006), *gh*-overexpression in fish from the F0104 line was able to increase 31% in weight and 11.4% in standard length compared to non-transgenic siblings. These findings reinforce that transgenesis for *gh* leads to a higher level of energy required to support body growth. In addition, GH exerts its physiological actions by binding to its cognate receptor in target tissues, and such interaction depends on its affinity and the density of the receptor on the cell membrane (Ranke and Wit, 2018). In this context, we first investigated whether *gh* overexpression could alter the expression of its receptor in intestinal cells. Intestinal mRNA expression of *ghrb* was significantly increased in *gh*-transgenic zebrafish. The expression of both *ghra* and *ghrb* in intestine was reported in many fish species (Jiao et al., 2006; Li et al., 2007; Fuentes et al., 2008). Di Prinzio et al. (2010) showed that both *ghr* paralogs were predominantly expressed in the intestine and liver of adult zebrafish. In parallel, *in situ* hybridization analysis revealed that *ghrb* transcript was localized in the zebrafish intestine at 30 h postfertilization (hpf) (Di Prinzio et al., 2010) that suggests the role of GH in the development of this tissue. In addition, the metabolic state and the availability of external nutrients play a role in regulating GHR expression (Bertucci et al., 2019). Collectively, these results indicate that GH promotes the gene expression of its receptor to enhance tissue responsiveness to the circulating GH under particular physiological circumstances.

The GI tract has been reported as a major target organ of IGF action (Bortvedt and Lund, 2012). Additionally, a considerable amount of evidence indicates that IGF1 is the main mediator of GH enterotrophic effects (Bortvedt and Lund, 2012; Ge et al., 2015). One of the most prominent effects is the stimulation of intestinal epithelial cell proliferation and maintenance of cell survival by reduction of apoptosis (Kuemmerle, 2012). We also identify a significant increase of i*gf1a* mRNA expression in the *gh*-transgenic intestine. Thus, we propose that excess circulating GH can transcriptionally regulate genes related to growth factors in the intestine, such as local production of IGF1 by enterocyte (Ge et al., 2015), to enhance the intestinal length and mass, and consequently improve absorptive plasticity of this tissue to meet the energy demand for accelerated growth. In this sense, Ohneda et al. (1997) demonstrated that the effects of IGF1 overexpression on the intestinal length and mucosal mass were similar to the effects of GH overexpression observed in this study.

Phenotypic flexibility of the GI tract in response to changes in demand of organisms has been described in a variety of avian and mammalian species [for a review, refer Starck (2003)] and in fish (Leigh et al., 2017). In this study, we investigate whether the chronic excess of GH could change the intestinal morphology and ultrastructure in the F0104 line. *Gh*-transgenic zebrafish presented an increase of 8% in intestinal length and 47% in intestinal mass with a higher ESI. These findings are in agreement with those described for salmon (Stevens et al., 1999; Stevens and Devlin, 2000, 2005) and mice (Ulshen et al., 1993), which proposed that excess GH promotes restructuring of the intestinal architecture. A longer intestine means that more digesta can be processed per unit time, which is consistent with higher feed intake (Leigh et al., 2017). Thus, the elongation of the intestine in fish overexpressing GH may be a necessary morphophysiological response to supply the high-energy demand induced by the excess of circulating GH.

In this study, the increase in intestinal mass in *gh*-transgenic zebrafish is due to the increase in the length and width of the intestinal villi and the increase in the height of the enterocytes. This absorption function is increased by the expansion of the intestinal surface from the increase of villi at the tissue level and the increase of microvilli at the cellular level (Wang et al., 2010), and not the cellular apical surface. In humans, villi and microvilli together amplify the small intestinal surface area by 60–120 times (Helander and Fändriks, 2014). Thus, we suggest that overexpression of *gh* can expand nutrient absorption capacity, extending the intestinal mucosal area, and providing dietary nutrients for growth maintenance.

In this study, we do not identify any difference in the number of intestinal folds and goblet cells or the microvilli surface area between *gh*-transgenic and non-transgenic counterparts. Studies with GH-transgenic salmon have reported an increase in the number of intestinal folds and absorptive surface area in the foregut of transgenic fish (Stevens et al., 1999; Stevens and Devlin, 2000, 2005). In those studies, the methodology used for the morphometric assessment of the intestine differed from the methodology used in the present study. Furthermore, the number or length of the intestinal microvilli was not considered for absorptive surface area calculations. The F0104 *gh*-transgenic zebrafish display larger villi and enterocytes, adjusting to a model of hypertrophy that is less costly from an energy point of view than producing a larger number of cells as reported by Starck (2003). This is likely a strategy to save energy for the maintenance of the intestine itself and to increase the function of this tissue in providing energy for somatic growth.

Among the many functions attributed to GH, its role in protein metabolism is important in the allocation of acquired nutrients toward anabolic processes (Leggatt et al., 2009; Reindl and Sheridan, 2012). In this sense, we analyze whether *gh-*transgenesis can modulate the expression of peptide transport in the intestine. The results show that transcription of *slc15a1a* and *slc15a1b* genes, which code for the PEPT1 isoforms, is induced in the intestine of *gh*-transgenic fish. Recently, Petro-Sakuma et al. (2020) demonstrated that the levels of intestinal *pept1a* and -*1b* transcripts were significantly increased in hypophysectomized tilapia (*Oreochromis mossambicus*) after GH replacement, indicating that this hormone could stimulate gut-specific transporters that underlie nutrient absorptive capacities. Consistently, the PEPT1 protein has been strongly associated with differentiated and mature absorptive enterocytes (Romano et al., 2014). Additionally, mice lacking intestinal PEPT1 has decreased intestinal uptake and dipeptide permeability (Hu et al., 2008), and also reduced energy intake and differences in the small intestinal morphology, independent of the diet (Kolodziejczak et al., 2013). Taken together, these observations lead us to suggest a potential interaction between enterotrophic effects of the GH-IGF1 system in improving nutrient absorption through transporters such as PEPT1.

Peptide Transporter 1 has emerged as one of the most studied nutrient transport systems in fish, especially due to the strong evidence of its involvement in compensatory growth (Verri et al., 2017). In European sea bass (*Dicentrarchus labrax*), a carnivorous species, Tevora et al. (Terova et al., 2009) reported a reduction in intestinal PEPT1 expression during fasting followed by an increase during refeeding. In zebrafish, PEPT1 mRNA expression increased approximately eight times higher in refeeding after short-term food deprivation (Koven and Schulte, 2012). These results suggest that this transporter operates directly for fish growth (Verri et al., 2011; Koven and Schulte, 2012). In support of this, when intestinal PEPT1 was inactivated in the nematode *Caenorhabditis elegans*, animals showed a severely retarded development, reduced progeny, and body size (Meissner et al., 2004). In addition, proteome analysis performed in worms lacking PEPT1 revealed downregulation of ribosomal proteins, leading to impaired ribosome biogenesis and reducing protein *de novo* synthesis (Geillinger et al., 2014; Spanier and Rohm, 2018). Transport of di or tripeptides involves the same amount of energy as transport of single free amino acids (Daniel, 2004). Also, it has been proven that short peptides are absorbed faster than free amino acids (Ostaszewska et al., 2010a, b). In this context, we suggest that *gh*-transgenic fish have a more efficient peptide transport to meet their metabolic demands and amino acid supply required by support anabolic process. We also hypothesize that the increased expression of PEPT1 in the intestine of *gh*-transgenic zebrafish may be a compensatory effect given that there were no differences in the surface area of the microvilli between the groups.

In fish, as in mammals, energy homeostasis and food intake are regulated by the interaction of central and peripheral (intestine, liver, etc.) endocrine pathways that respond to the energy status of the entire body and requirements (Gorissen et al., 2006; White et al., 2016). Regarding food intake, it has been reported that *gh*-transgenic fish eat much more due to uncontrolled appetite regulation through changes in the GH axis (Devlin et al., 2001; Raven et al., 2006; Dalmolin et al., 2015; White et al., 2016) and that the intestinal length plus maximum intestinal distension would determine the intake of digestible nutrients and energy (Raven et al., 2006; Leigh et al., 2017). The current study findings for the *gh*-transgenic zebrafish agree with the previous studies with respect to their need to satisfy daily energy needs by the significant increase of feed intake relative to non-transgenic counterparts. Moreover, it is well known that intestinal morphology and the overall expression and function of nutrient transporters in enterocytes are triggered by the dietary composition and nutrient supply (Spanier, 2014; Spanier and Rohm, 2018). Therefore, in agreement with Stevens and Devlin (2005), we also believe in the increased intestinal size and absorption capacity of *gh*-transgenic zebrafish as a result of the direct and indirect effects of excess GH.

In the current study, *gh*-transgenic zebrafish ingested large amounts of protein as a consequence of higher feed intake. Cyprinid culture is one of the largest protein production businesses worldwide and aquaculture is one of the fastest-growing industrial sectors in the world. Understanding the regulation and functionality of nutrient transporters present in the GI tract of fish can contribute to the development of strategies to increase the commercial production of domesticated fish (Daniel, 2004; Verri et al., 2017; Petro-Sakuma et al., 2020). In particular, the PEPT1 transporter can be an interesting target for genetic engineering with the objective of developing new fish lines with better use of nutrients present in the feed and, consequently, better growth performance.

In conclusion, this study has added to the understanding of how GH supports somatic growth in a *gh*-transgenic zebrafish line. Together, the results suggest that *gh*-overexpression stimulates the expression of its receptor in the intestine with a consequent local production of IGF1, which can act directly to increase the mass and absorption area of this organ. In addition, *gh*-overexpression promotes hyperphagia that provides a greater amount of nutrients in the intestinal lumen and stimulates the synthesis of di-tripeptide transporters, which are the main route of absorption of protein degradation products after a meal. Although the signaling pathway by which GH regulates amino acids and peptide transporters is still unknown, PEPT1 function can be integrated into the physiological response scheme of the *gh*-transgenic model to enhance peptide uptake.

## Data Availability Statement

The raw data supporting the conclusions of this article will be made available by the authors, without undue reservation.

## Ethics Statement

All animal handling procedures were approved by the “Ethics Committee of the Federal University of Rio Grande (FURG),” Protocol number 23116.008403/2018-32.

## Author Contributions

MM, BN, TS, and MK contributed to the conception and design of the study. MM, BN, TS, MK, CC, and JR developed the experiments and performed statistical analysis. VP and LR helped in the process of standardization and analyses of histological sections. MM, CC, and BN contributed to the pictures design. MM wrote the first draft of the manuscript. LM provided review and editing, project administration, supervision, and funding acquisition. All authors contributed to revising the manuscript, reading, and approving the submitted version.

## Conflict of Interest

The authors declare that the research was conducted in the absence of any commercial or financial relationships that could be construed as a potential conflict of interest.

## Publisher’s Note

All claims expressed in this article are solely those of the authors and do not necessarily represent those of their affiliated organizations, or those of the publisher, the editors and the reviewers. Any product that may be evaluated in this article, or claim that may be made by its manufacturer, is not guaranteed or endorsed by the publisher.

## Footnotes

1
https://www.zfin.org
